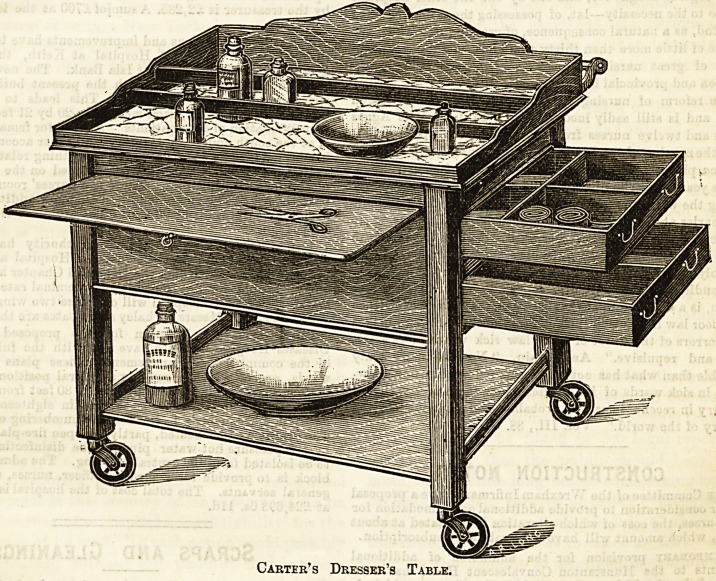# Hospitals of the World

**Published:** 1893-03-18

**Authors:** 


### HOSPITALS OF THE WORLD.*

### THE PROGRESS OF ENGLISH NURSING.

Whatever may have been the case as regards hospital construction, England cannot claim a foremost place in the history of nursing. The abolition of the religious orders at the Reformation deprived the sick poor of the skilled attendance which had been theirs in many a convent and monastery through the country. The persons whom it was possible to secure for attendance on the sick in the newly founded hospitals were often gathered from the lowest classes, received scant remuneration and no training whatever. At St. Bartholomew's, in Henry the Eighth's reign, a Matron with twelve sisters under her was appointed under the revised secular system to attend to the sick. "What nursing was in those days may be gathered from the fact that these twelve nurses were ordered, when their daily work among their patients was done, to occupy themselves in spinning, sewing, mending of sheets and shirts, or some other virtuous exercise such as they should be appointed unto. They are also wisely commanded, in common with the Matron and most of the other officers, if they should perceive anything amiss, to inform the governors and meddle no further therein."

* "Hospitals and Asylums of the World," 4 vols., and Portfolio of Plans. (Messrs. Churchill and the Scientific Press. 1893.)

"Before the year 1840 nurses were almost the worst set of working women; the only points to be settled on engaging a nurse were that she was not Irish and not a confirmed drunkard. 'We always engage them without any character,' wrote a doctor, 'as no respectable person would undertake so disagreeable an office.' Every vice was rampant among these women, and their aid to the dying was to remove pillows and bed-clothes, and so hasten the end."

In the year 1840 the first start was made. "Mrs. Fry and Lady Inglis in that year founded the first nursing institution in London at Osnaburgh Square, under the patronage of Queen Adelaide, and at the suggestion of Dr. Gooch and Robert Southey. Its members were called 'nursing sisters,' and they were paid for their services. The institute still flourishes in new premises in Devonshire Square."

A few years later the establishment of two Anglican Sisterhoods, the Society of the Holy Trinity at Devonport, and St. Mary at Wantage, both adopting the care of the sick as part of their work, greatly helped to change the tone of general opinion towards the duties of a sick nurse, and restore the dignity and sacredness lent them by the words of the Great Master, "Inasmuch as ye have done it unto one of the least of these My brethren, ye have done it unto Me." In 1854 two other Sisterhoods, that of St. John the Baptist at Clewer, and the Nursing Sisters of St. Margaret, East Grinstead, lent themselves to the same work. Even from those least in sympathy with their tenets the work of these and other Anglican Sisterhoods must meet with due recognition: Firstly, in rendering it possible for ladies to devote themselves once more to duties at that time carried through under the most trying and almost prohibitory conditions; secondly, in developing a trained school of nurses by whom the nursing art has been largely advanced; thirdly, and perhaps most remarkable, in readily adapting themselves to the best modern requirements of training and

Carter's Dresser's Table.

organisation. It must not be forgotten also that from Clewer and Wantage the first English nurses went out to India, where the nursing had been previously entirely in the hands of Ayahs and ward-boys.

But the work of the Sisterhoods was necessarily slow, and was carried on in the strictest privacy of which it would admit. It remained for Miss Nightingale to bring before the world the nursing profession as a vocation for women, demanding the best material, and calling forth the highest qualities. Her work in the Crimean War, in conjunction with some of the sisters of St. John's House, was an object lesson in nursing for Europe. But for England it resulted in something more. With the £40,000 subscribed for her by the nation, the Nightingale School, in connexion with St. Thomas's Hospital, was founded in 1840, for the training of nurses, and an incalculable impetus was thus given to nursing as a profession. For the nurses thus trained were eagerly sought for, and one by one the other hospitals awoke to the necessity—1st, of possessing the skilled labour; and 2nd, as a natural consequence, of creating it. Thus, the course of little more than thirty years has seen the establishment of great nursing schools in connection with every London and provincial hospital.

The reform of nursing in workhouse infirmaries began later, and is still sadly incomplete. "In 1865 Miss Agnes Jones and twelve nurses from St. Thomas's Hospital undertook the nursing of the Liverpool Workhouse Infirmary, and became pioneers in the scientific nursing of sick paupers. Three years later she died of typhus fever, the first martyr among the workhouse nurses." Some few of the poor law infirmaries at the present time are model institutions, in no respect inferior to the best hospitals. Yet it is possible to write: "In those sick wards where there is inadequate, or possibly no nursing at all in any proper meaning of the term, the condition of the patients, especially when helpless and infirm, is a disgrace to our civilization. The very excellence of a poor law institution such as Marylebone Infirmary makes the horrors of the worst of poor law sick wards more startling and repulsive." And again, "Nothing more truly horrible than what has actually occurred within our knowledge in sick wards of infirmaries under the poor law in this country in recent times has probably ever happened in the history of the world." Vol. III., 88.

## Figures and Tables

**Figure f1:**